# Could the bulbar urethral end location on the cystourethrogram predict the outcome after posterior urethroplasty for pelvic fracture urethral injury?

**DOI:** 10.1080/2090598X.2022.2138119

**Published:** 2022-11-01

**Authors:** Ahmed M. Harraz, Adel Nabeeh, Ramy Elbaz, Abdalla Abdelhamid, Mohamed Tharwat, Amr A. Elbakry, Ahmed S. El-Hefnawy, Ahmed El-Assmy, Ahmed Mosbah, Mohamed H. Zahran

**Affiliations:** Urology and Nephrology Center, Mansoura University, Mansoura, Egypt

**Keywords:** Posterior urethra, posterior urethroplasty, pelvic fracture urethral injury, cystourethrogram, stricture urethra

## Abstract

**Objectives:**

To identify cystourethrogram (CUG) findings that independently predict the outcome of posterior urethroplasty (PU) following pelvic fracture urethral injury (PFUI).

**Methods:**

Findings of CUG included the location of the proximal end of the bulbar urethra in zones A (superficial) or B (deep) according to its relationship with the pubic arch. Others included the presence of pelvic arch fracture, bladder neck, and posterior urethral appearance. The primary outcome was the need for reintervention either endoscopically or by redo urethroplasty. Independent predictors were modeled using a logistic regression model and a nomogram was constructed and internally validated using 100-bootstrap resampling. Time-to-event analysis was performed to validate the results.

**Results:**

A total of 196 procedures in 158 patients were analyzed. The success rate was 83.7% with 32 (16.3%) procedures requiring direct vision internal urethrotomy, urethroplasty, or both in 13 (6.6%), 12 (6.1%), and 7 (3.6%) patients, respectively. On multivariate analysis, bulbar urethral end located at zone B (odds ratio [OR]: 3.1; 95% confidence interval [CI]: 1.1–8.5; p = 0.02), pubic arch fracture (OR: 3.9; 95%CI: 1.5–9.7; p = 0.003), and previous urethroplasty (OR: 4.2; 95% CI: 1.8–10.1; p = 0.001) were independent predictors. The same predictors were significant in the time-to-event analysis. The nomogram discrimination was 77.3% and 75% in the current data and after validation.

**Conclusions:**

The location of the proximal end of the bulbar urethra and redo urethroplasty could predict the need for reintervention after PU for PFUI. The nomogram could be used preoperatively for patient counseling and procedure planning.

## Introduction

Posterior urethroplasty (PU) for pelvic fracture urethral injury (PFUI) is a challenging procedure and has been considered ‘a specialty within a specialty’ [1]. The reported success rate ranged from as low as 60% up to 91% [2–8]. Consequently, the search for a tool to predict the complexity and the outcome of the procedure is mandatory to optimize patient counseling and preoperative planning [9].

In PFUI, the distraction occurs at the bulbo-membranous junction, which ultimately leads to the upward, lateral, or posterior displacement of the prostatic urethra. Hence, the complexity of the procedure commonly stems from the identification of the posterior urethra, the length of the urethral defect, and the presence of bladder neck (BN) injury; all requiring ancillary maneuvers to overcome the distraction defect [9–12]. Herein, the assessment of the BN and posterior urethra is of utmost importance for preoperative surgical planning as well as anticipating the complexity of the procedure.

Combined antegrade and retrograde cystourethrogram (CUG) is the mainstay radiological modality for the assessment of patients with PFUI undergoing PU [13,14]. Although the need for BN and posterior urethral assessment is invaluable for surgical planning, it is not uncommon that the posterior urethra could not be visualized during CUG. Bladder neck and posterior urethral visualization require the patient relaxes the BN and gentle trial of voiding which is not consistent in every patient. Therefore, a proportion of patients with CUG might have missed data about the posterior urethra. Although antegrade flexible cystoscopy provides the status of BN, it does not determine neither the depth of the posterior urethra nor its direction of displacement. Nevertheless, it is an essential step to be performed before attempting the procedure as the approach of repair will be determined accordingly. It was suggested that tadalafil could be used to relax the BN before the CUG [15].

On this basis, we examined the utility of CUG, regardless of the appearance of BN, as a potential predictor of PU outcome excluding patients with BN injury diagnosed by flexible cystoscopy. The focus of this study is on the bulbar urethral location on the CUG. We hypothesized that as deep as the anterior urethra proximally extends into the pelvis, it would require more deep surgical dissection, and an increase in urethroplasty complexity is anticipated which potentially might affect the outcome. This depth of the bulbar urethral extension could be postulated by reviewing the CUG images. Hence, we evaluated the CUG findings and other clinical data and demonstrated their impact on the success rate after PU. Using this tool to predict the outcome of the procedure would be of utmost importance being done for every patient and easily interpreted by the urologist.

## Methods

### Study design

A retrospective review of patients’ electronic records who underwent PU for PFUI between January 2002 and December 2017 in a tertiary referral center was performed. The study received the appropriate institutional review board approval with no informed consent because of the retrospective nature.

Only patients with good quality CUG images were included by confirming the complete appearance of one obturator foramen and the obliteration of the other indicating that the whole length of the urethra was visible [16]. In this position, the depth of the bulbar urethral extension into the pelvis could be assessed. Patients with BN avulsion identified by antegrade flexible cystoscopy or those with bulbar urethral necrosis were excluded.

### Combined antegrade and retrograde cystourethrogram (CUG)

All CUG images performed immediately before surgery, were reviewed by a dedicated single uro-radiologist. The technique of the CUG involved the positioning of the patient in a supine oblique position (35–45 degrees) which can be confirmed by a closed downward oriented obturator foramen. Through the suprapubic catheter, the bladder is gradually filled under gravity with a diluted contrast agent and then the patient is asked to notify when he has an urge to micturate. A trial of voiding could allow for a better proximal assessment of the BN and prostatic urethra. Then a retrograde urethrogram is performed.

### Posterior urethroplasty (PU)

Antegrade cystoscopy is routinely performed before PU to identify the BN and posterior urethra. All procedures were done based on a standardized approach by the dedicated urethral reconstruction team. Technical tips included adequate mobilization of the bulbar urethra, and corporeal splitting and/or inferior pubectomy were performed whenever appropriate to achieve a tension-free mucosa-to-mucosa anastomosis. A urethral catheter was fixed for 3 weeks. Postoperatively, patients were reviewed at 3 monthly intervals during the first year and once yearly thereafter. Follow-up consisted of IPSS score, uroflowmetry, retrograde urethrogram, and urethroscopy if there was a suspicion of re-stenosis. Patients reported outcomes included IPSS score, IIEF scores were recently introduced but not involved in the current study.

### Measurements

**Figure 1.**
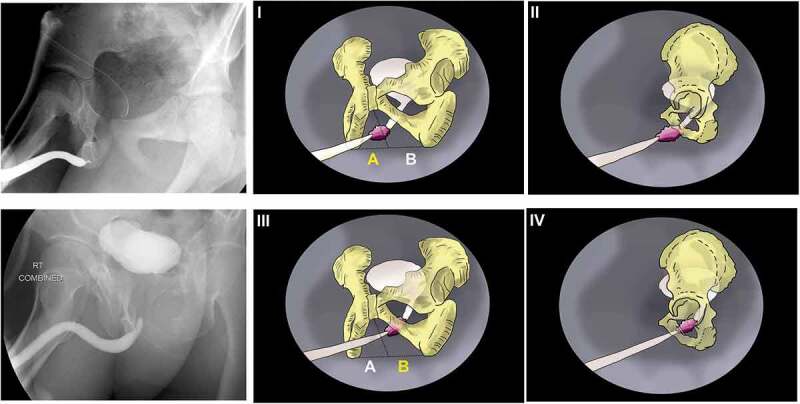
The zones of the prostate. I: posteroanterior view showing the location of the distal end of the bulbar urethra in zone A. II: Lateral view demonstrated assumed minimal fibrosis and a possibly better outcome. III: posteroanterior view showing the location of the distal end of the bulbar urethra in zone B. IV: Lateral view demonstrated the need for deeper dissection and more extensive fibrosis and possibly worse outcomes.

For this study, the BN and posterior urethra appearance in the CUG was reported but not included in the analysis. The relationship between the proximal end of the bulbar urethra in relation to the pubic arch ‘pubis symphysis (PS) and pubic rami’ was assigned a location either in zone A or zone B (Figure 1). Zone A is located distal to an imaginary line extending from the lower border of PS to a point midway between the two ischial tuberosities. On the other hand, zone B is located proximal to that line. When the bulbar urethral end location is in zone A, we assume that the pelvic injury was more superficial, minimal dissection is expected and a better outcome is awaited. On the other hand, zone B lies deep in relation to the pubic arch; hence, the urethral injury is anticipated to be deep in the pelvis and extensive dissection is expected and that might be reflected in the complexity of the surgery and the outcome.

In addition to bulbar urethral location, the presence of apparent pelvic displacement/fracture in the pubic arch including PS diastasis was recorded. The BN was described as not visualized, a dimple, or completely visualized posterior urethra. Lastly, the bulbar urethral to defect ratio was determined in patients with a visualized posterior urethra [17].

### Study outcome

The primary outcome of the study was to identify preoperative data and radiological findings from CUG that independently predict the failure rate after PU for PFUI. The failure was defined as the need for further instrumentation after surgery either by direct vision internal urethrotomy (DVIU) and/or redo urethroplasty. These factors are used to construct a nomogram for calculating the probability of the failure. The secondary outcomes included a time-to-event analysis to account for the effect of time. To avoid multiple testing on the same patients, a sensitivity analysis based on the number of patients and not the number of procedures was conducted.

### Statistical analysis

Comparison of continuous variables was performed using the student t-test and categorical variables by the Chi-square test or Fischer’s exact test whenever indicated. A logistic regression model determined the independent predictors on multivariate analysis. Coefficients of the model were used to construct the nomogram. The validation of the nomogram included discrimination which is measured using the area under the curve (AUC). The AUC and calibration plots were provided for the original dataset and after 100-bootstrap resampling. The clinical usefulness of the nomogram was evaluated using the decision curve analysis (DCA) that was validated after 10-fold cross-validation. The analysis was repeated after including only the last urethroplasty performed in patients with multiple procedures and considering earlier surgeries as positive history. For the time-to-event analysis, Kaplan Meier survival curves were performed based on the time of failure or last follow-up. The log-rank test was used for the detection of statistical significance. Statistical analysis was performed using IBM SPSS version 25 and R programing language v. 3.6.3 (http://www.r-project.org) with packages rms, rmda, and riskRegression.

## Results

### Demographics and perioperative data

Electronic records were available for 250 patients of whom 158 (196 urethroplasty procedures) had CUG images available and of adequate quality. The mean patients’ age and body mass index were 32.7 (14.2) years and 27.7 (8.1) Kg/m2, respectively. The procedure was done 2 times in 26 (16.5%) patients and 3 times in 6 (3.8%). Primary realignment was not done in any of our patients with all underwent suprapubic cystostomy at the time of urethral injury. The median (IQR) time to urethroplasty was 4 (3–6) months. Postoperatively, superficial wound infection occurred in 4 patients and required frequent dressing. Blood transfusion was required in 7 patients. The median (IQR) hospital stay was 6 days (5–9).

In the CUG, the BN was not visualized in 76 (38.8%) studies while zones A and B were found in 85 (43.4%) and 111 (56.6%), respectively. Pubis symphysis diastasis and pubic ramus fracture were apparent in 12 (6.1%) and 94 (48%), respectively. Zone B was significantly associated with redo urethroplasty procedures [30 (27%)] compared to zone A [13 (15.3%)] (p = 0.04). Similarly, zone B was found in 50 (45%) procedures requiring ancillary maneuvers (corporeal splitting/pubectomy) compared to zone A which was present in 27 (31.8%) procedures, with borderline statistical significance (p = 0.05). No supra-crural rerouting was documented in this cohort of patients.

### Outcome analysis

**Figure 2.**
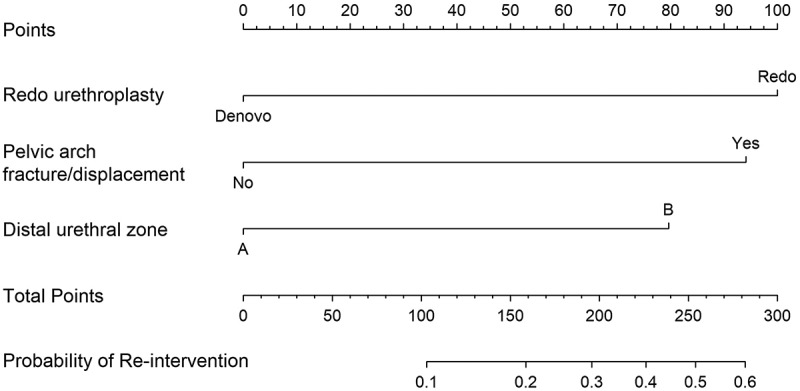
Nomogram constructed from factors significantly predicted the need for reintervention after posterior urethroplasty for pelvic fracture urethral injury. For instance, a patient with a history of urethroplasty had a pubic arch fracture and the distal urethral end is in zone B would be assigned points (Points scale) 100 + 95 + 80 = 275 (total points scale) which corresponds to 0.6 probability of reintervention (Probability of re-intervention scale).

The success rate was reported in 164 (83.7%) procedures with 32 (16.3%) required DVIU, urethroplasty, or both in 13 (6.6%), 12 (6.1%), and 7 (3.6%) patients, respectively. The median (IQR) time to recurrence was 5 (3–10) months. The bulbar urethral end location at zone B was significantly associated with failure [26 (23.4%) vs 6 (7.1%), p = 0.002]. A history of previous urethroplasty [15 (34.9%) vs 17 (11.1%), p < 0.001] and the presence of pubic arch fracture/displacement [24 (25.5) vs 8 (7.8), p = 0.002] were also significantly associated with stricture recurrence (Table 1). On multivariate analysis, previous urethroplasty (odds ratio [OR]: 4.2; 95% confidence interval [CI]: 1.8–10.1; p = 0.001), bulbar urethral end at zone B (OR: 3.1: 95%CI: 1.1–8.5; p = 0.02), and pubic arch fracture (OR: 3.9; 95%CI: 1.5–9.7; p = 0.003) were independent predictors. The nomogram describing the multivariate analysis is shown in Figure 2.

**Table 1.** Cystourethrogram findings in posterior urethroplasty procedures performed for pelvic fracture urethral injuries.

Variable		Success (n = 164)	Failure (n = 32)	p-value
Bladder neck	Not visualized	66 (86.8%)	10 (13.2%)	0.08
	Dimple	28 (93.3%)	2 (6.7%)	
	Patent posterior urethra	70 (77.8%)	20 (22.2%)	
Pubic arch fracture/ displacement	No	94 (92.2%)	8 (7.8%)	0.002
	Yes	70 (74.5%)	24 (25.5%)	
Distal urethral zone	A	79 (92.9%)	6 (7.1%)	0.004
	B	85 (76.6%)	26 (23.4%)	
Bulbar urethral to defect ratio*	Median [IQR]	3.4 [2, 5.5]	3.2 [2, 5.5]	0.8

The analysis is per procedure (n = 196) as many patients underwent more than one procedure.

*The ratio cannot be calculated in 106 patients. It is calculated using the percentage of bulbar urethral length to defect length.

### Time-to-event analysis

**Figure 3.**
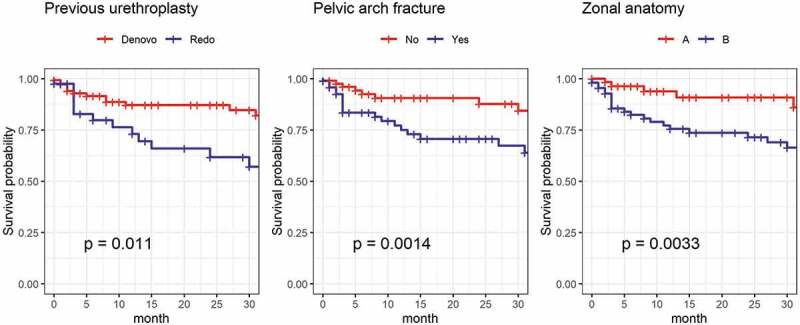
Kaplan Meier curves for the predictors of posterior urethroplasty outcome. P-values are calculated using the log-rank test.

Significant predictors were previous urethroplasty: denovo vs redo (12 months: 87% vs 73%, 24 months: 87% vs 62%, p = 0.01), respectively, pubic arch fracture: No vs yes (12 months: 90% vs 75%, 24 months: 88% vs 71%, p = 0.001), respectively, zonal anatomy: A vs B (12 months: 94% vs 75%, 24 months: 91% vs 71%, p = 0.003), respectively. Results are displayed in Figure 3.

### Sensitivity analysis

The analysis was repeated after including only the last procedure performed in patients with multiple procedures and omitting early surgeries form the analysis. Recurrence occurred in 22 (14%) patients. Significant variables included previous urethroplasty (p = 0.02), pelvic arch fracture (p = 0.001), and distal urethral zone anatomy (p = 0.02) (Table 2). On multivariate analysis, independent predictors were redo urethroplasty [OR (95%CI): 3.4 (1.2:9.5), p = 0.02], and the presence of pubic arch fracture [OR (95%CI): 5.2 (1.8:17.3), p = 0.003] while zone B was not statistically significant [OR (95%CI): 2.5 (0.9:8.6), p = 0.09].

**Table 2.** Predictors of failure in patients who underwent urethroplasty for pelvic fracture urethral injury.

Variables		Success (n = 136)	Failure (n = 22)	p-value
Previous urethroplasty	Denovo	109 (80.15%)	12 (54.55%)	0.02
	Redo	27 (19.85%)	10 (45.45%)	
Pubic arch fracture/displacement	No	84 (61.76%)	5 (22.73%)	0.001
	Yes	52 (38.24%)	17 (77.27%)	
Zonal anatomy	A	71 (52.21%)	5 (22.73%)	0.02
	B	65 (47.79%)	17 (77.27%)	

The analysis is per patient (n = 158) considering only the last urethroplasty performed in patients with multiple urethroplasties.

### Nomogram evaluation

**Figure 4.**
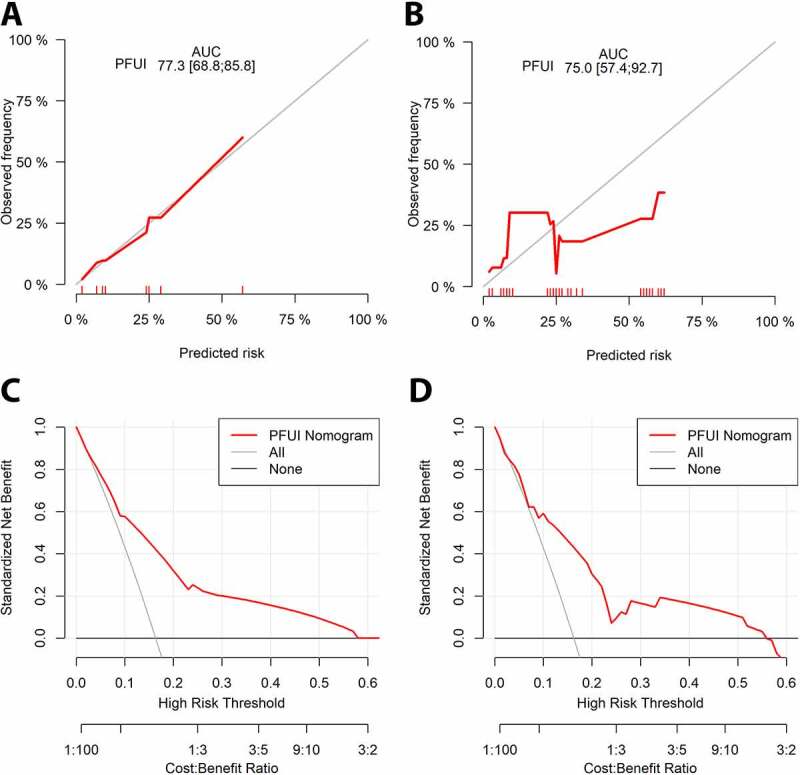
Calibration plots in the original data (A) and after 100-bootstrap resampling (B). The closer the red curve to the reference curve, the better the calibration of the nomogram. Decision curve analysis (DCA) with the net benefit (NB) (the difference between true and false positives divided by the sample size) plotted against a range of threshold probabilities in the original dataset (C) and after a 10-fold cross-validation (D). The NB is better than treating-all or treating-none in both settings. AUC: Area under the curve; PFUI: Pelvic fracture urethral injury.

The discrimination of the nomogram in the original data was 77.3% and after 100-bootstrap resampling was 75%. Calibration plots in the original data and after internal validation are demonstrated in Figure 4. Calibration plots delineate the relationship between the nomogram predicted probabilities of failure and the actual events. Decision curve analysis demonstrated better net benefit than treating-all and treating-none in both the original data and after 10-fold cross-validation. Curves are depicted in Figure 4.

## Discussion

Although visualizing the BN and posterior urethra is an essential step that can be cystoscopically accomplished, the main drive to conduct this study was that the BN and posterior urethra were not apparent in a significant proportion of our patients who underwent CUG and consequently we were not able to implement it as a consistent variable in all patients. In addition, with the exclusion of patients with BN injury and bulbar urethral necrosis, we hypothesized that even if the posterior urethral dislocation is extensive, information from the distal urethra would help in drawing the geographic picture of the urethral pathology.

We have shown that the location of the proximal end of the bulbar urethra as well as the presence of pubic arch fractures, in addition to the history of urethroplasty were the independent predictors. The constructed nomogram is simple and easy to use preoperatively to facilitate patients counseling and adequate preoperative planning. The importance of these results stems from the availability and easy interpretation of the CUG images in all patients undergoing PU.

In the current study, the location of the proximal end of the bulbar urethra was an independent determinant of the failure rate regardless of the position or the length of the posterior urethra. In the vast majority of cases, the prostatic urethra and BN are hypothesized to be normal except on rare occasions such as in pediatric patients [16]. Conversely, the presence of the proximal end of the bulbar urethra superficially in zone A would indicate a more superficial injury, and less dissection is required to remove all scarred tissue compared if the urethral end is in zone B where the dissection should progress deeply in the pelvis. We have used an imaginary line in the center of a plane that is equivalent to the anatomical site of the perineal membrane between the pubic rami in the normal setting. This plane is arbitrary and is suggested as an objective tool to describe the depth of the bulbar urethra in relation to the pubic arch.

A similar finding has been delineated by Fu et al [18]. In an analysis of 376 patients, they found that strictures with the proximal end closer to the BN (<3 cm), were four-fold more likely to fail compared with strictures far (>3 cm) from the BN (success rate: 64.6% vs 90.5%: p < 0.001). The authors further confirmed our hypothesis that strictures that were closer to the BN required extensive dissection and tissue removal, had poor exposure, and had very limited space for urethral instrumentation which might negatively reflect on stricture recurrence [18].

To avoid misinterpretation of the CUG findings, one important distinction should be noted. First, if the bulbar urethral end is in zone A but there is apparent kinking, adequate length, and anatomical deviation, this might indicate a severe injury that resulted in a hematoma pushing the bulbar urethra distally and locating it in zone A which is not the case as our hypothesis. On the other hand, if the above-mentioned signs are not visualized, this might indicate less severe injury and fulfilling our hypothesis.

The presence of pubic arch fracture was shown to independently predict the need for reintervention in the current study. The types of pelvic fracture that are commonly associated with urethral injury included fractures of all 4 pubic rami (straddle fractures), fractures of inferior pubic ramus with a widened PS, and deep ring fracture-dislocations (Malgaigne’s fractures) [16]. Therefore, pubic arch fractures are possibly constant findings in patients with PFUI. This might explain that if these fractures are not apparent in the images of CUG, this would indicate less severe injuries and consequently associated with less degree of scarring.

Likewise, a history of previous urethroplasty is suggested to increase the likelihood of reintervention. Recurrent stenosis after urethroplasty is significantly associated with more severe scarring and devitalized tissues secondary to the previous dissection. In 50 patients with previous failed repair, the success rate was 75% [19]. In a recent systematic review of literature, the success rate of PU for recurrent strictures varied from 69–100% [20]. It is to be noted that these surgeries might require additional ancillary procedures including supra-crural rerouting and Mitrofanoff appendicovesicostomy. Nevertheless, none of our patients required additional procedures beyond inferior pubectomy. In addition, in cases of severe extensive fibrosis and the proximal end of the urethra was not identified, the urethra was anastomosed to the prostate anteriorly provided that the BN was intact.

The relation between preoperative imaging and the complexity and success rate of PU has been previously described. Koritum has measured the bulbar urethral length as well as the urethral gap and had proposed an index [17]. His results have shown that a urethral gap that is more than one-third of the bulbar urethra would most likely require an elaborated rather than a simple perineal approach. Likewise, the gapometry score has been suggested to predict the approach in 38 children undergoing PU [21]. The authors have shown that the gapometry score significantly predicted the surgical approach utilized. Nevertheless, it is obvious that the posterior urethra must be visualized to estimate the gap length which is not the case in our cohort where the posterior urethra was not apparent in approximately half of our patients. In addition, the hypothesis behind this gapometry score depends on the elasticity of the bulbar urethra to overcome the urethral gap and the extent of bulbar urethral dissection which is variable based on the surgeon’s preference.

A posterior urethral stenosis score was postulated based on urethrography images with other parameters [9]. This score utilized the cause, location, length, presence, or absence of fistula to predict the complexity and failure after surgery. Although being statistically significant, the score necessitated both ends of the urethra to be visualized to estimate the urethral gap. In a similar attempt, Horiguchi and associates investigated the role of preoperative magnetic resonance imaging (MRI) in the prediction of intraoperative complexity in 74 patients who underwent PU for PFUI [22]. The authors have found that a narrow pubo-urethral stump angle predicted the need for maneuvers beyond the urethral mobilization. Albeit the results were encouraging, and in concordance with others [23], the use of MRI for the evaluation of PFUI remains an issue of cost-effectiveness and the added benefit would not change the practice as long as similar knowledge could be obtained from the CUG [24].

We applied the analysis to all procedures performed in every patient, so we achieved the highest number of CUG findings to be evaluated as procedure findings. Nevertheless, this may cause some statistical confusion as procedures performed in the same patient has similarities regarding patients’ demographics. To resolve this, only unique features of the CUG were selected for the analysis. In addition, to validate the results, a secondary analysis was performed based on the patient and considering only the last urethroplasty performed, which confirms our results but with border-line non-significant zonal anatomy which might be attributed to the small outcome size based on the reduction of the cohort number.

The study has several limitations that deserve mention. Initially, the study is retrospective and only patients with available CUG images of adequate quality were included. Consequently, the incidence of treatment failure described in this report might not represent the true incidence in the whole series. However, the previously reported success rate was centered approximately around 80%-90% [8,10,14,18], which is in concordance with this study. The number of patients achieving the required outcome is relatively small which might reduce the power of the study. Furthermore, some important factors that might affect the success rate were not investigated such as the level of the surgeon expertise albeit all surgeons had special training in urethral reconstruction. It is important to acknowledge that a proper description of the outcome would include patients reported outcome measures that are not available in this study. Continence status, erectile and ejaculatory functions are better characterized through a prospective longitudinal study.

In conclusion, the signs detected by the CUG could predict the probability of reintervention after PU for PFUI. The location of the proximal end of the bulbar urethra, pubic arch fractures, and history of previous urethroplasty were the independent predictors. The constructed nomogram could be beneficial preoperatively in patients’ counseling and preoperative planning.

## Figures and Tables

**Table:** 

AUC	Area under the curve
BN	Bladder neck
CUG	cystourethrogram
DCA	decision curve analysis
DVIU	Direct vision internal urethrotomy
IQR	Interquartile range
PFUI	pelvic fracture urethral injury
PS	pubis symphysis
PU	posterior urethroplasty
